# Supplementary material for: A high-resolution mRNA expression time course of embryonic development in zebrafish
Source: eLife. 2017 Nov 16;6:e30860. doi: 10.7554/eLife.30860 (PMC5690287; doi:10.7554/eLife.30860)
Supplement: Supplementary file 6. [file elife-30860-supp6.zip › biolayout-clusters-files/Cluster228-genes.html]

Cluster228


# Cluster228: Genes

| Ensembl ID | Gene Name | Chr | Start | End | Biotype |
| --- | --- | --- | --- | --- | --- |
| ENSDARG00000101523 | ENSDARG00000101523 | 21 | 40647989 | 40653375 | protein\_coding |
| ENSDARG00000004447 | aplnr2 | 7 | 38414494 | 38418304 | protein\_coding |
| ENSDARG00000008174 | hoxb1a | 3 | 23638350 | 23640594 | protein\_coding |
| ENSDARG00000021232 | nkx2.7 | 8 | 50296213 | 50299303 | protein\_coding |
| ENSDARG00000097203 | si:ch211-176l24.4 | 25 | 17752523 | 17756544 | protein\_coding |
| ENSDARG00000007077 | si:dkey-145p14.5 | 9 | 33049409 | 33064822 | protein\_coding |
